# Parenteral Vaccination With a Tuberculosis Subunit Vaccine in Presence of Retinoic Acid Provides Early but Transient Protection to *M. Tuberculosis* Infection

**DOI:** 10.3389/fimmu.2019.00934

**Published:** 2019-05-03

**Authors:** Antonella Riccomi, Giovanni Piccaro, Dennis Christensen, Carla Palma, Peter Andersen, Silvia Vendetti

**Affiliations:** ^1^Department of Infectious Diseases, Istituto Superiore di Sanità, Rome, Italy; ^2^Notified Body, Istituto Superiore di Sanità, Rome, Italy; ^3^Department of Infectious Diseases Immunology, Statens Serum Institute, Copenhagen, Denmark

**Keywords:** retinoic acid, mucosal immunity, mucosal immunization, parenteral immunization, adjuvant, mucosal adjuvant, *M. tuberculosis*

## Abstract

Most microbes invading through mucosal surfaces cause disease and therefore strategies to induce mucosal immune responses are strongly needed. Vitamin A metabolites, such as retinoic acid (RA), play crucial roles in programming T and B cells to home to mucosal compartments, therefore we evaluated the capacity of RA to elicit mucosal immune responses against tuberculosis (TB) after parenteral vaccination. We found that mice immunized through subcutaneous injections with the TB subunit vaccine (CAF01+H56) in presence of RA show enhanced mucosal H56-specific IgA responses and enhanced Ag-specific CD4^+^ T lymphocytes homing to the lung as compared with control mice. Immunization with CAF01+H56 in presence of RA resulted in lower bacterial loads in the lungs of mice 14 days after challenge with virulent Mycobacterium tuberculosis (Mtb) as compared to mice immunized in the absence of RA or vaccinated with BCG. Higher amounts of IFNγ and IL-17 pro-inflammatory cytokines were found in lung homogenates of mice immunized with CAF01+H56 and RA 24 h after Mtb infection. However, 6 weeks after infection the protection was comparable in vaccinated mice with or without RA even though treatment with RA during immunization is able to better contain the inflammatory response by the host. Furthermore, at later stage of the infection a higher percentage of Mtb specific CD4^+^PD1^+^ T lymphocytes were found in the lungs of mice immunized with CAF01+H56 and RA. These data show that an enhanced mucosal immune response is generated during parenteral vaccination in presence of RA. Furthermore, RA treatment contained the bacterial growth at an early stage of the infection and limited the inflammatory response in the lung at later time points.

## Introduction

Most of viruses, bacteria, parasites or allergens invades the hosts through mucosal tissues. The immune system has developed specific mucosal defenses to avoid invasion and contain the infection of potentially harmful microbes. To strengthen mucosal responses is important for preventing and containing infectious diseases (1). It has been demonstrated in animal models and in humans that mucosal immunization induces effective humoral and cellular immune responses both at mucosal surfaces and at systemic levels (2–4). In particular the nasal route of immunization is the most efficient at inducing immune responses locally, at distant mucosal sites and systemically (5–7). However, there are many restraints using the nasal route, which are related to the potential toxicity of the mucosal adjuvants and the delivery of vaccines (8–10). Several adjuvants and delivery systems have been suggested for developing nasal vaccines (2, 11, 12), however an effective and safe mucosal adjuvant to be used in human has not yet been released. Novel strategies to induce mucosal immune responses are strongly needed.

Retinoic acid (RA) is a vitamin A metabolite able to modulate tolerance and immunity at mucosal sites (13, 14). RA regulates CD4^+^ T cell differentiation toward Th1/Th17 polarization (15, 16) and controls DCs homeostasis at lymphoid and mucosal sites (17, 18). Furthermore, antigen presentation in presence of RA imprints mucosal homing capacity on T and B cells (18–20). Although implicated in gut homing, RA induces the homing of antigen-specific T cells at other mucosal compartments including vagina and lungs (21). However, the mechanisms underlining homing to extra-intestinal mucosal compartments of antigen-specific T cells induced by RA are not well described.

It has previously been suggested that protection against infection with Mtb correlates with the capacity of T cells to home to the lungs (22–25) and that vaccination with the subunit vaccine CAF01+H56 induces T lymphocytes able to home to lung parenchyma (23). Here, we evaluate whether subcutaneous vaccination with CAF01+H56 in presence of RA will cause increased homing of H56-specific T cells to the lung and ultimately protection against TB challenge.

We found that parenteral vaccination with CAF01+H56 in presence of RA induces mucosal H56-specific IgA and Mtb-specific CD4^+^ T lymphocytes to the lung. Vaccination in presence of RA provides early but transient increased protection to Mtb infection and it is able to better contain the inflammatory response by the host at later time point. In parallel with the progression of the disease an increased percentage of antigen-specific T cells with an exhausted-like phenotype appeared in the lung of mice vaccinated in presence of RA.

## Materials and Methods

### Media and Reagents

RPMI 1640 medium supplemented with 2 mM L-glutamine, 1% (v/v) nonessential amino acids, 1% (v/v) Sodium-pyruvate, 55 μM 2-mercaptoethanol, 100 U/ml penicillin, 100 μg/ml streptomycin and 10% FBS (Euroclone S.p.a. Italy), was used as complete medium in all cultures. Two different media were used for the culture of Mtb: a liquid medium necessary for the culture of bacteria (Broth Dubos Tween Albumin—DTA, Difco, Detroit, USA), and a solid one for the count of bacteria present in lung and spleen at the end of the experiments (Middlebrook 7H10 agar with 10% OADC enrichment and 0.25% glycerol). Ovalbumin protein (OVA) was purchased from Sigma-Aldrich, USA, H56 was provided by Staten Serum Institute, SSI, Denmark in the contest of the European project ADITEC and H56 CD4-specific peptides p63 (H-QDAYNAAGGHINAVFN-OH), p67 (H-THSWEYWGAQLNAMK-OH), and H56 CD8-specific peptides p28 (H-MPVGGQSSF-OH), p43 (H-IYAGSLSAL-OH), and H-AIOGNVTSI-OH were obtained from JPT Peptide Technologies GmbH, Germany. All trans Retinoic Acid (RA) and sesame oil were purchased from Sigma-Aldrich, USA. CAF01 was provided by Staten Serum Institute, SSI, Denmark in the contest of the European project ADITEC.

### Bacterial Cultures

*M. tuberculosis* H37Rv (ATCC 27294, American Type Culture Collection, Manassas, VA) was grown at 37 °C in Broth Dubos Tween Albumin—DTA, Difco, Detroit, USA), under agitation (120 rpm). For *in vivo* experiments, bacteria in mid-log growing phase (OD600 0.2–0.4), were centrifuged (3,000 rpm, 30 min) and re-suspended in RPMI-1640 supplemented with 5% heat-inactivated FBS and 2 mM L-Glutamine. Aliquoted stocks were stored at −80°C until use. Bacterial stock concentrations were determined by CFU assay.

### Parenteral Immunization and Mycobacterium Infection

Groups of 10 female CB6F1 mice aged 6 to 8 weeks were treated 1 day before and 1 day after each immunization with 300 μg/dose/day of RA or with its vehicle (sesame oil) the first time, 200 μg/dose/day the second time and 100 μg/dose/day the third time by subcutaneous injection (100 μg/dose). The immunization with CAF01 (125 μg/dose) and OVA protein (5 μg/dose) or H56 (0.5 μg/dose) every 21 days for three times by subcutaneous route at the base of tale in 200 μl. Groups of mice treated or untreated only with RA were included as controls. A group of mice immunized with subcutaneous injection of 5 × 10^5^ CFU of BCG was included as control. Four weeks after last immunization, mice were infected i.v. in a lateral tail vein with 10^5^ CFU/mouse of virulent strain of Mtb (H37Rv, 27294 obtained by ATCC) re-suspended in 200 μl of PBS. Handling of infected material was performed in a biosafety level three facility, mice were housed in an isolator cages. A sample of Mtb H37Rv was thawed sonicated for 15 seconds and diluted in PBS containing glass bearings at concentration of 10^5^/200 μl.

### Ethical Statement

Animals were maintained in the animal facilities at the Istituto Superiore di Sanità (ISS) under specific pathogen-free conditions, according to European Union guidelines and Italian legislation (Decreto Legislativo 26/2014). Animal studies were authorized by the Italian Ministry of Health and reviewed by the Service for Animal Welfare at ISS (Authorization n. 82014-b of 15/01/2014). Animals were euthanized with minimal suffering and discomfort at the end of the experiments by CO_2_ inhalation using approved chambers.

### Samples Collection

Serum and mucosal fluids from vaginal washes were collected 2 weeks after each immunization. Serum was obtained from blood collected from retro-orbital plexus of anesthetized mice and stored at −20°C until assayed. Vaginal washes were obtained by using 150 μl of phosphate-buffered saline (PBS), introducing 50 μl for three times into the vaginal of mice using a Gilson pipette.

### Analysis of Antibody Isotypes

Serum and vaginal washes were tested by a standard enzyme-linked immunoassorbant assay (ELISA). Ninety-six well plates (Greiner bio-one, Germany) were coated with OVA protein (5 μg/well) or H56 (0.5 μg/well) overnight at 4°C. Plates were washed with PBS containing 0.05% (v/v) Tween 20 (Sigma Aldrich, USA), and blocked for 2 h with 200 μl of PBS containing 1% Bovine Serum Albumin (BSA, Sigma-Aldrich, USA), dilutions of sera and vaginal washes from each mice were added to wells and incubated 2 h at RT. The plates were washed and biotin-conjugated goat anti-mouse IgG, IgA, IgG1, or IgG2a (Southern Biotech, USA), respectively, diluted 1:20000, 1:20000, 1:5000, 1:2000 in PBS-BSA-Tween 20 were added to the wells and kept 2 h at RT. Plates were washed and horse radish peroxidase HRP-conjugated streptavidin (ANASPEC, Belgium) diluted 1:20000 in PBS-BSA-Tween20 was added and kept 30 min at RT. After washing with PBS-Tween20, the reaction of Ag-antibody was measured by using the 3.3, 5.5–TetraMethylBenzidine substrate (TMB, SurModics, USA). The reaction was blocked after 5 to 15 min with 50 μl of 0.2 M H_2_SO_4_. Titers were determined as the reciprocal of the highest dilution with an absorbance of more than 0.3 units (for IgG) and 0.2 units (for IgA) compared to the negative controls.

### Evaluation of Antibody Secreting Cells

The frequency of Ag-specific antibody secreting cells (ASC) was evaluated in cells isolated from bone marrow of mice by an enzyme-linked immunospot (ELISPOT) assay. Briefly, 2 × 10^5^ cells/well were incubated over night at 37°C in PVDF 96-well plates (Millipore) previously coated with OVA (20 μg/ml) or H56 (5 μg/ml). Plates were washed and incubated with HRP-conjugated goat anti-mouse IgA antibody (Southern Biotech, USA) followed by Amino Ethyl-Carbazole chromogenic substrate (AEC- Sigma Chemicals, Co., St. Louis, MO, USA). Spots were evaluated using an automated reader (A.EL.VIS, Hanover, Germany).

### Isolation of Murine Lung Lymphocytes

Lung cells were isolated according to the protocol described in Lung Dissociation kit (Miltenyi Biotec, USA) with some modification. Lungs were carefully removed from thoracic cavity, washed in cold PBS and dissected into single lobes. The lobes were then digested in RPMI 1640 10% (v/v) FCS, 25 mM Hepes (Sigma-Aldrich, USA), 300 U/ml Collagenase IV (Worthington Biochem, USA) and 20 U/ml DNAse I (Roche, Germany) and dissociated using gentleMACS Dissociator™. Samples were incubated 30 min at 37°C under continuous rotation and further homogenized by gentleMACS Dissociator™. Cells suspensions were then filtered through a 100 μM-pore-size nylon cell strainer (BD, USA), re-suspended in 35% Percoll (Sigma-Aldrich, USA), layered under a 80% Percoll gradient, and then centrifuged to isolate the lymphocyte enriched population at the 35–80% Percoll interface.

### Cytokines Detection

Lung homogenates were filtered through membranes (pore size 0.22 μm, Millipore, Malsheim, France) and analyzed for IFN-γ, IL-17, IL-1β, IL-6, and MIP-1β by a specific quantitative sandwich ELISA kit (mouse Quantikine, R&D System, USA), in accordance with the manufacturer's instructions. Quantification was made according to a standard curve obtained for each cytokine standard provided by manufacturer.

### Flow Cytometry

To assess the phenotype, cells were stained with specific labeled antibody diluted 1:30 in 1% BSA-PBS for 15 min at 4°C. Antibodies used were anti-CD4-PECY7 (clone GK1.5) anti-CD69-FITC (clone H1.2F3) anti-CD45.2- FITC (clone 104) Tetramers-APC (Ag85B-FQDAYNAAGGHNAVF) anti-PD1-PE (clone J43). Then cells were washed two times in PBS and re-suspended in 100 μl of PBS containing paraformaldehyde 4% and incubated overnight to allow the neutralization of Mtb, if present. Then cells were washed two times in PBS and acquired on a FACSCalibur™ instrument running CellQuest software and analyzed by FlowJo software.

### Intravascular Staining

A total of 3 μg of anti-CD45.2-FITC (clone 104) diluted in 200 μl of PBS were injected intravenously (i.v.) into a mouse via the tail vein. Three minutes later, the animals were sacrificed. The spleen and lung were harvested and lymphocytes were isolated as described. Immunofluorescence staining was performed as described.

### Enzyme-Linked Immunospot (ELISPOT) Assay

The number of IFN-γ-secreting cells and IL-5-secreting cells in PBMC preparations was evaluated by the BD™ ELISPOT mouse IFN-γ and IL-5 ELISPOTSet (BD Biosciences Pharmingen), in accordance with the manufacturer's instructions. Briefly, 96 well nitrocellulose plates were coated with 5 μg/ml of the purified anti-mouse IFN-γ or with 5 μg/ml of the purified anti-mouse IL-5. Cells (3 × 10^5^/well) were cultured in complete RPMI, incubated with CD4 or CD8 H56 peptides (2 μg/ml), ConA (5 μg/ml), or media alone. After 24 h for IFN-γ and 48 h for IL-5 at 37°C, cells were removed and the wells were incubated with biotinylated anti-mouse IFN-γ or IL-5 monoclonal Ab, and with streptavidin-HRP-conjugate. Cytokines producing cells were visualized by addition of the AEC substrate (Sigma-Aldrich, USA) and reaction was stopped by washing with water. Spots were counted using ELISPOT reader (A.EL.VIS GmbH, Hanover, Germany).

### Colony Forming Units

To determine bacillary loads in the tissues, the left lobe of the lung and the spleen were homogenized using gentleMACS™ Dissociator (Milteny Biotec) in sterile PBS and 10-fold dilutions prepared in distilled water, were plated on Middlebrook 7H10 agar with 10% OADC enrichment (Becton Dickenson) and 0.25% glycerol. Colony-forming units (CFU) were enumerated following incubation at 37°C for 21 days.

### Statistical Analysis

For statistical analysis Microsoft Excel (Microsoft Corp., Redmond, WA) and Prism 6.0 GraphPad software (San Diego, CA) were used. Data are expressed as the mean ± standard error of mean (SEM). One-way ANOVA test was performed followed by Tukey's or Student's *t*-test to evaluate the significance. Statistical significance was considered with *p* < 0.05.

## Results

### Parenteral Vaccination in Presence of Retinoic Acid Promotes Antigen-Specific Antibody Responses at Systemic and Mucosal Sites

**Figure 1:**
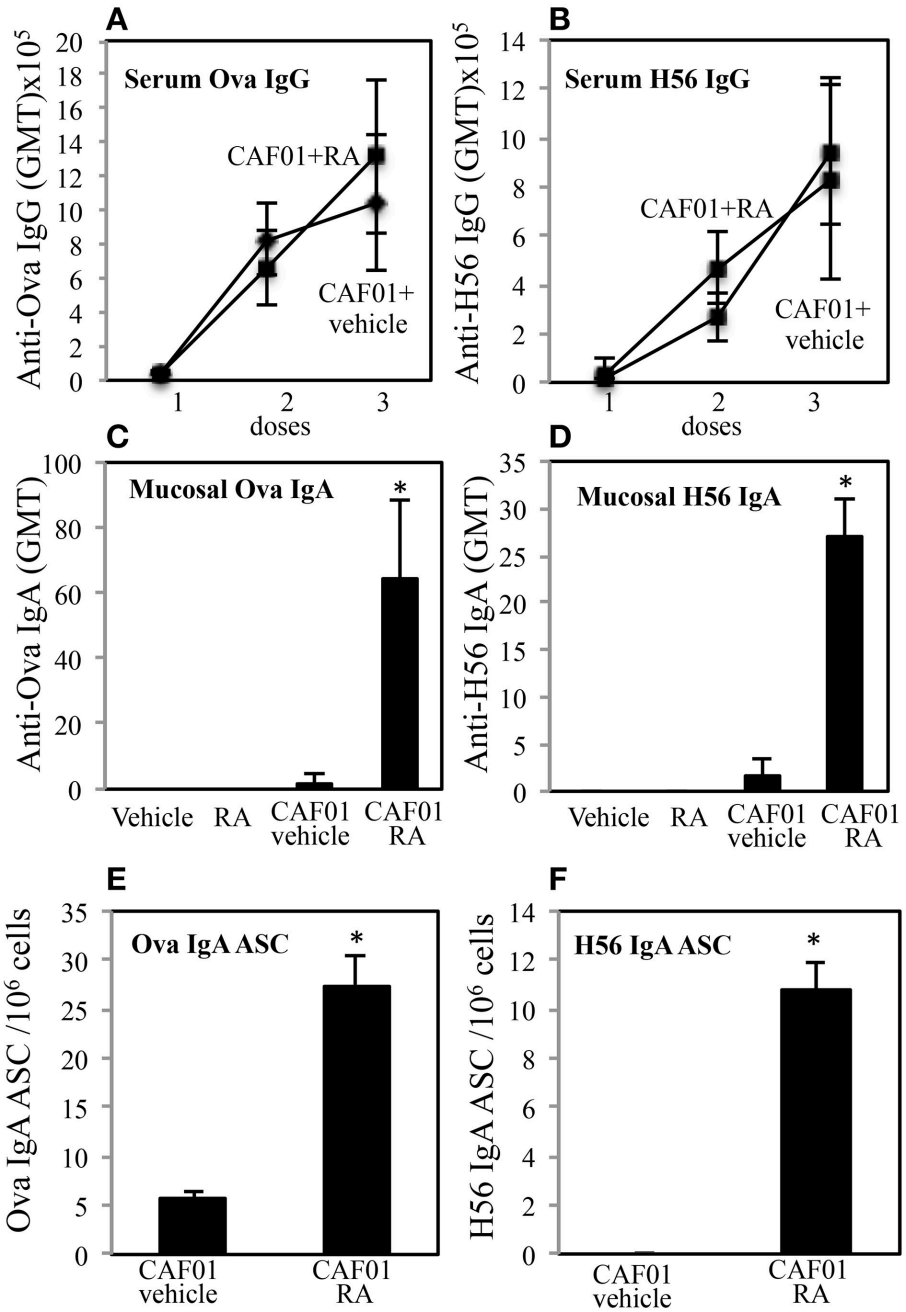
Parenteral immunization in presence of RA promotes mucosal Ag-specific immune response in CB6F1 mice. Serum IgG **(A,B)** and vaginal IgA **(C,D)** responses to OVA (5 μg/dose) **(A,C)** and to H56 (0.5 μg/dose) **(B,D)** in CB6F1 mice (5/group) treated or untreated with RA (300 μg/dose, 200 μg/dose and 100 μg/dose) or its vehicle (sesame oil) and immunized with CAF01 (125 μg/dose) and OVA protein (5 μg/mouse) or H56 (0.5 μg/dose). Results are expressed as IgG or IgA geometric mean titer (GMT). The IgG titer was determined in sera and IgA in vaginal washes. Ag-specific long living plasma cells in the bone marrow of CB6F1 mice treated or untreated with RA or its vehicle (sesame oil) and immunized with CAF01 (125 μg/dose) and OVA (5 μg/dose) **(E)** or H56 (0.5 μg/dose) **(F)**. Ag-specific IgA antibody secreting cells (ASC) were analyzed by ELISPOT assay. Bars indicate SEM of 4 mice /group. Asterisks indicate significant differences between groups at the analyzed time point; *p-value < 0.05. One rapresentative experiment is shown of three performed.

To evaluate the ability of RA to elicit both mucosal and systemic immunity by conferring to systemic adjuvants the capacity to induce mucosal immunity, experiments with CAF01 used as adjuvant and OVA or the mycobacterial H56 fusion protein as antigens (Ags) were performed. Groups of CB6F1 mice were immunized by subcutaneous injection at the base of the tail with CAF01 (125 μg/dose) and OVA protein (5 μg/dose) or H56 (0.5 μg/dose) three times, 3 weeks a part. Mice were treated 1 day before and 1 day after each immunization with RA (300 μg/first dose, 200 μg/second dose, 100 μg/third dose) or with its vehicle (sesame oil). Serum OVA and H56-specific IgG antibody responses were examined 2 weeks after each immunization and mucosal OVA and H56-specific IgA responses were measured 2 weeks after last immunization in the vaginal washes. Strong systemic Ag-specific IgG response was generated following the immunizations, as previously reported (26, 27) and no significant differences were observed between mice treated with RA or its vehicle at systemic level (Figures 1A,B). However, higher OVA and H56 specific IgA titers were found in the vaginal washes of mice immunized in presence of RA (Figures 1C,D). In the groups of mice that received the Ags in the absence of adjuvant, the IgA and IgG titers were lower than 256. These data suggest that immune responses at mucosal sites are induced by parenteral vaccination in presence of RA.

To verify whether IgA secreting long living plasma cells were induced after parenteral immunization in presence of RA, OVA and H56-specific antibody secreting cells (ASC) were analyzed in bone marrow of mice 1 month after last immunization by an ELISPOT assay. Higher number of anti-OVA and H56-specific IgA ASC was found in cells isolated from bone marrow of mice treated with RA as compared to mice treated with RA vehicle (Figures 1E,F).

### The Quality of the Immune Response Induced by Vaccination Is not Affected by RA Treatment

**Figure 2:**
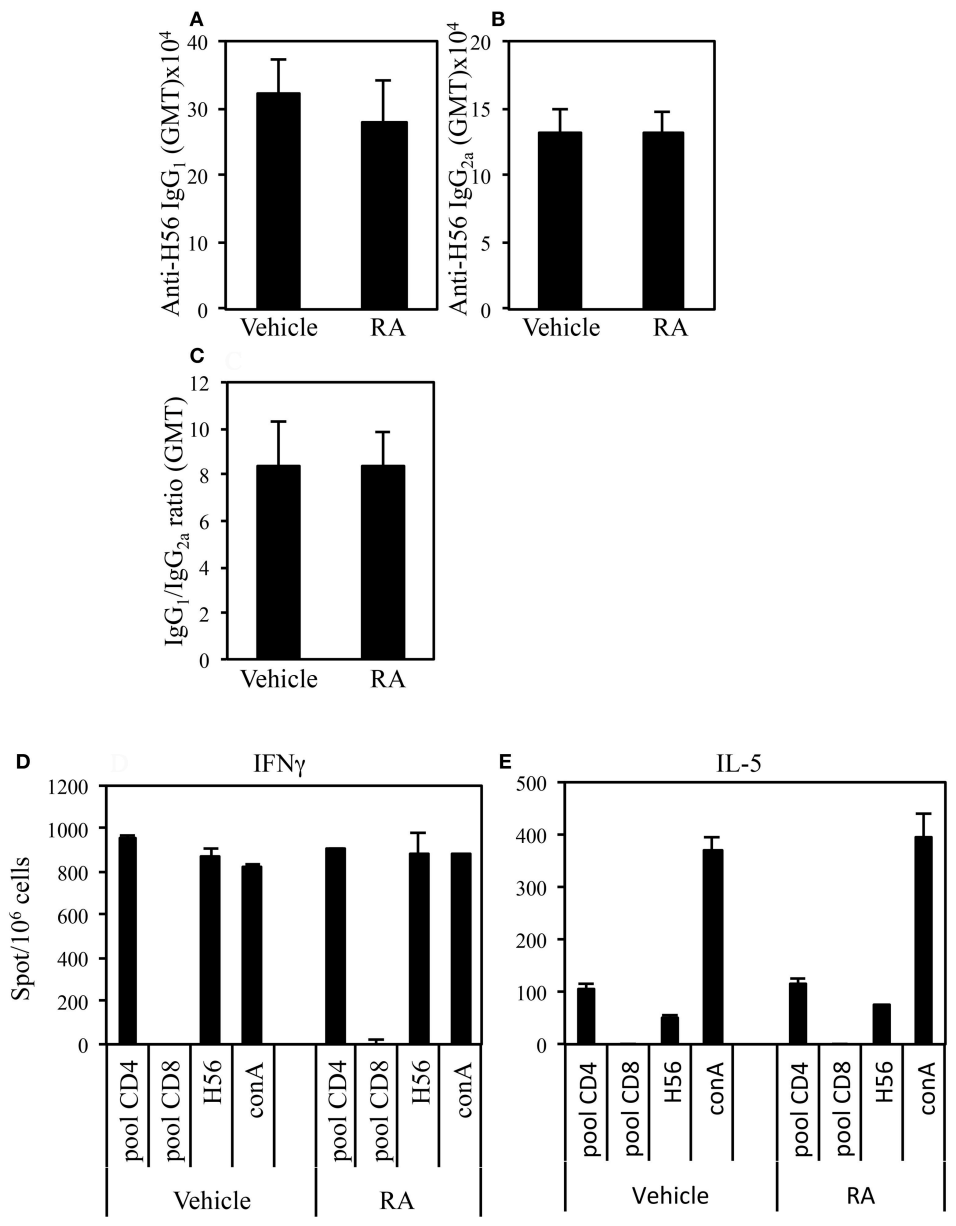
Serum levels of Ag-specific IgG1 **(A)** and IgG2a **(B)** antibodies from CB6F1 mice treated or untreated with RA (300, 200, and 100 μg/dose) or its vehicle (sesame oil) and immunized with CAF01 (125 μg/dose) and H56 (0.5 μg/dose). Values are indicated with geometric mean titer (GMT). **(C)** indicates the ratio between IgG1 and IgG2a titer. Bars indicate SEM. Ag-specific IFNγ **(D)** and IL-5 **(E)** responses measured in cells from peripheral blood by ELISPOT. Results are expressed in number of spots per 10^6^ of cells of CB6F1 mice treated or untreated with RA or its vehicle and immunized with CAF01 (125 μg/dose) and H56 (0.5 μg/dose). Bars indicate SEM.

Adjuvants can be classified on the basis of the quality of the immune response they are able to elicit. We asked whether the administration of RA in combination with a systemic adjuvant could change the quality of the immune response. The effect of RA administration in combination with CAF01 and H56 on the H56-specific IgG1 and IgG2a antibody responses and on the balance of the Th1/Th2 induced cytokines was evaluated. Following the immunization schedule described above, serum H56-specific IgG1 and IgG2a were evaluated in mice immunized in presence or absence of RA 2 weeks after last immunization. High levels of both H56-specific IgG1 and IgG2a were found in mice immunized and treated with RA or its vehicle with higher titer of the IgG1 subclass as compared to IgG2a (Figures 2A,B). Neither the antibody levels nor the ratio between IgG1 and IgG2a were changed (Figure 2C) in mice treated with RA compared to its vehicle. In addition, IFN-γ and IL-5 production was evaluated by ELISPOT assay in cells derived from peripheral blood of mice immunized in presence or absence of RA 1 month after last immunization. Cells were stimulated with a pool of H56-derived CD4 or CD8-specific peptides or with the whole protein H56. Comparable levels of IFN-γ (Figure 2D) and IL-5 (Figure 2E) were produced in cultures stimulated by a CD4-specific pool of peptides or whole H56 fusion protein. These data suggest that treatment with RA does not affect the quality of immune responses induced by immunization with CAF01+H56.

### Lung Colonization by H56-Specific CD4^+^ T Cells in Mice Systemically Immunized in Presence of RA

**Figure 3:**
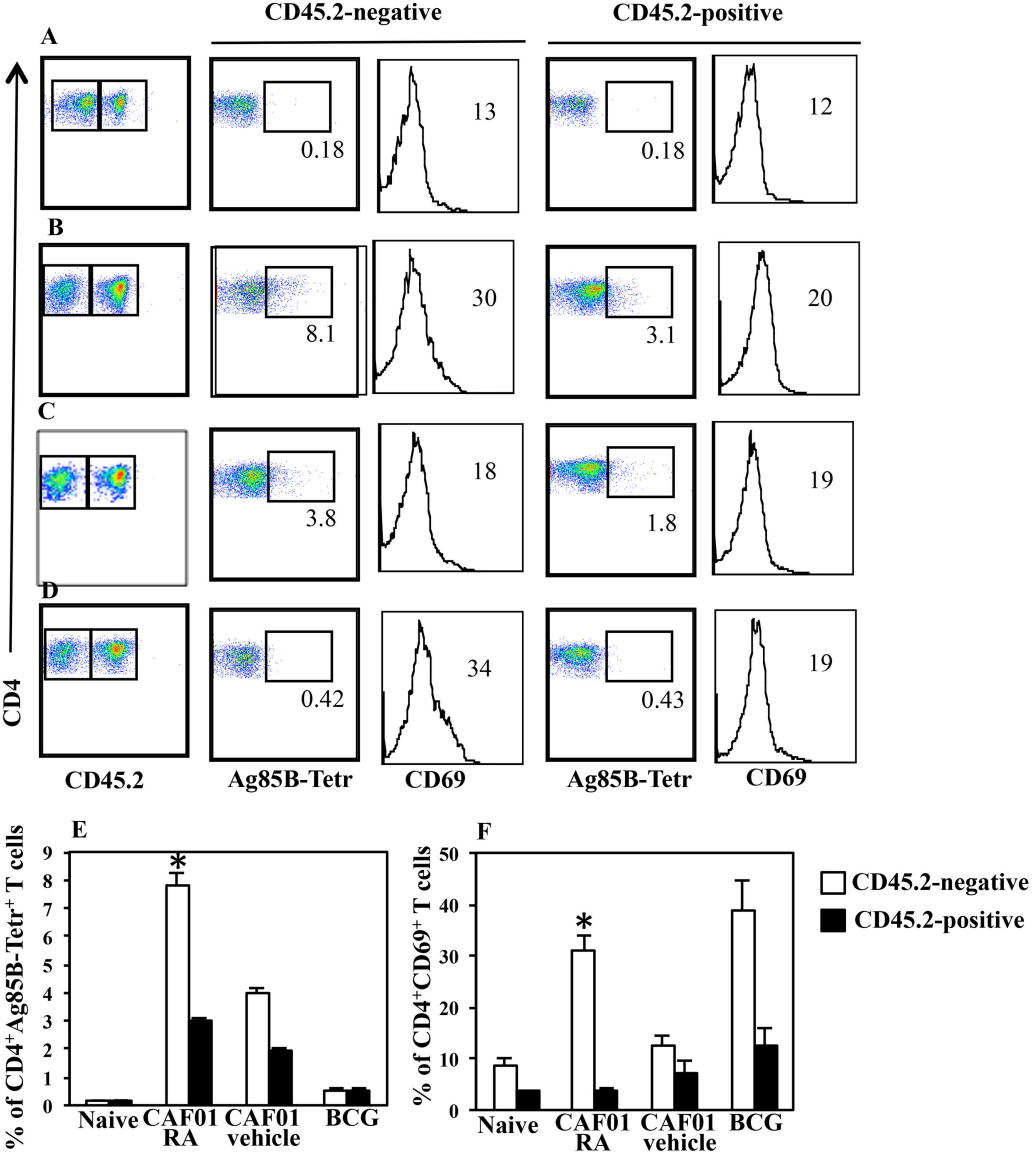
Lung colonization by H56-specific CD4^+^ T cells in mice parenterally immunized in presence of RA. Untreated naïve CB6F1 mice **(A)** or mice treated with RA **(B)** or its vehicle **(C)** were immunized with CAF01 (125 μg/dose) and H56 (0.5 μg/dose) or immunized with BCG **(D)** as tuberculosis vaccine control. One month after last immunization by combining intravascular (IV) anti-CD45 and *ex-vivo* Ag85B-tetramers and anti-CD69 staining, the percentage of Ag-specific CD4^+^ T lymphocytes was evaluated in the lung parenchyma (CD45.2-negative) and intravascular compartments (CD45.2-positive). The graphs show the percentage of CD4^+^Ag85B^+^ cells **(E)** and the percentage of CD4^+^CD69^+^ T cells **(F)** in the parenchyma and intravascular compartments. Bars indicate the SEM. *p-value < 0.05. One rapresentative experiment is shown of two performed.

Immunization with CAF01+H56 subunit vaccine promotes lung homing capacity of effector CD4 T cells that go through clonal expansion after Mtb challenge (23). Here, we evaluate how parenteral vaccination with CAF01 and H56 in presence of RA, impacts the colonization of lung compartments by H56-specific T cells. Mice were immunized following the immunization schedule described above or with BCG as tuberculosis vaccine control. One month after the last immunization by combining intravascular (IV) anti-CD45 and *ex-vivo* Ag85B-tetramers staining, we found a higher percentage of Ag-specific CD4^+^ T lymphocytes homing in both lung parenchyma and intravascular compartments in mice immunized and treated with RA as compared with untreated mice or mice immunized in presence of vehicle (Figures 3A–C,E). This correlates with a higher expression on parenchyma lung T lymphocytes of CD69, a marker preferentially expressed in tissue resident T cells. On the other hand, even if a high percentage of CD69^+^ T cells is found in the lung parenchyma of mice vaccinated with BCG (Figures 3D,F), low percentage of Ag85B-specific T lymphocytes was detected in both parenchyma and vascular lung compartment (Figures 3D,E). These data indicate that the parenteral immunization with CAF01+H56 in presence of RA further enhances the migration of Ag-specific T lymphocytes to the lung, suggesting that they could be effective against Mtb challenge.

### Vaccination With CAF01+H56 in Presence of RA Results in a Lower Bacterial Load in the Lungs 14 Days After Challenge With Virulent Mycobacterium Tuberculosis

**Figure 4:**
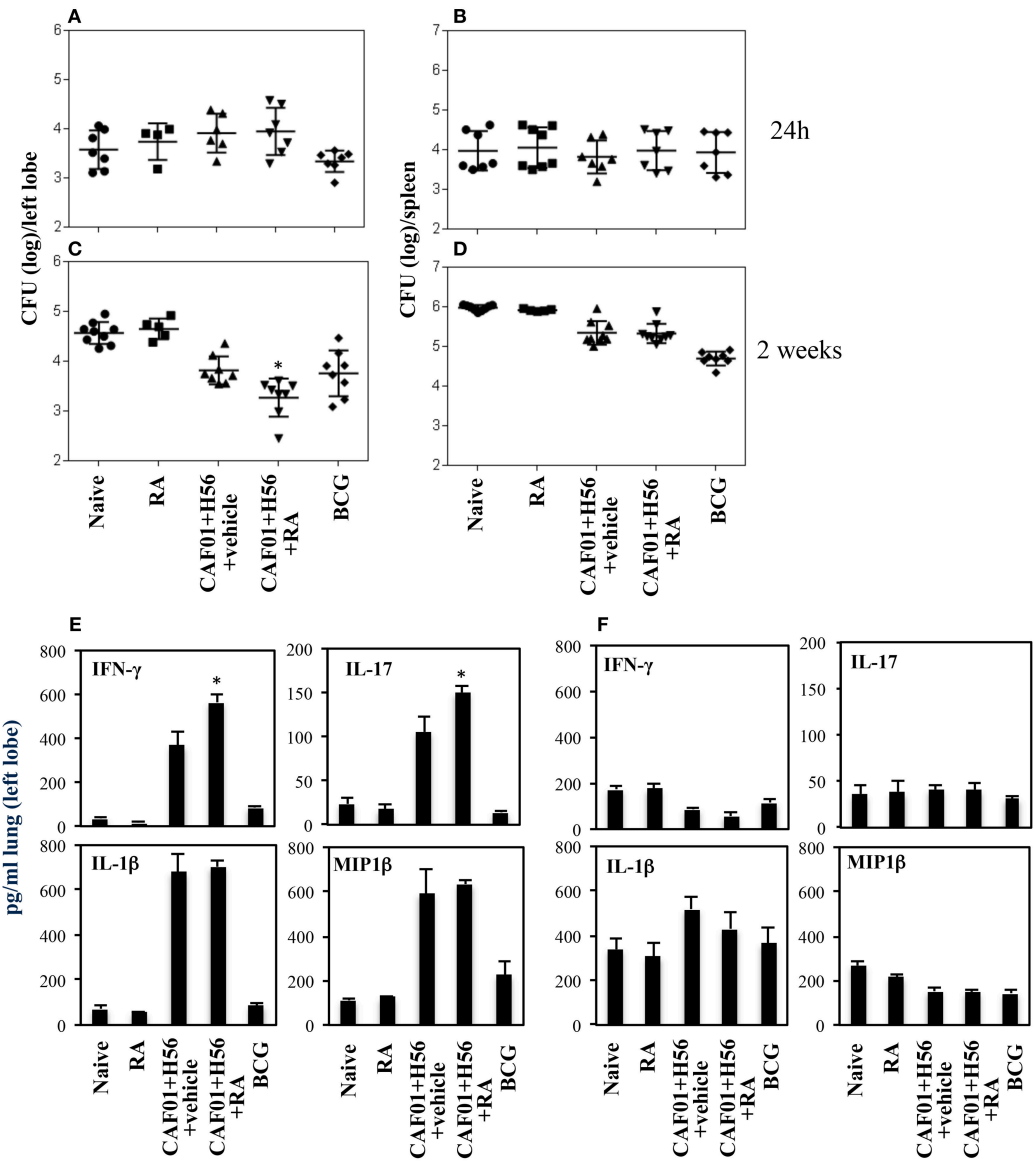
Colony forming unit (CFU) in the lung **(A,C)** and in the spleen **(B,D)** of CB6F1 mice. Mice (5–9/group) were treated or untreated with RA (300 μg/dose, 200 μg/dose and 100 μg/dose) or its vehicle, parenterally immunized with H56 (0.5 μg/dose) and CAF01 (125 μg/dose) and infected with Mtb (10^5^ CFU) 1 month after last immunization. Colony forming units were enumerated 24 h and 14 days in the lung **(A,C)** and in the spleen **(B,D)** after challenge. Results are expressed in log_10_ of the number of CFU/ml. Bars indicate SEM and stars (*) indicate that the differences between the group treated with RA and its vehicle are significant (*p* < 0.05). Pro-inflammatory cytokine production in the lungs of CB6F1 mice 24 h after Mtb infection. IFNγ, IL-17, IL-1β and MIP1β levels were analyzed by ELISA assay in the lung homogenates 24 h **(E)** and 14 days **(F)** after infection with Mtb in CB6F1 mice treated or untreated with RA or its vehicle and immunized with H56 (0.5 μg/dose) and CAF01 (125 μg/dose). One rapresentative experiment is shown of three performed.

The subunit CAF01+H56 vaccine confers protection by controlling the lung bacterial load in mice challenged with Mtb (28). To assess whether the administration of RA with the parenteral immunization with CAF01 and H56, could have any additional effects on the protection, mice vaccinated as described above, were intravenously infected with the virulent strain of Mtb (H37Rv) (10^5^ CFU/dose) 1 month after last immunization. Lung colonization by Mtb and bacterial growth were analyzed by evaluating the colony forming unit (CFU) in the lung and spleen 24 h, and 14 days after Mtb infection. Vaccination with BCG (5 × 10^5^ CFU/dose) performed once at the beginning of the immunization schedule was included as control. We found that 24 h after infection, mycobacteria colonize lung and spleen at comparable levels among the different groups (Figures 4A,B). The bacterial load progressively increased in the lung of un-vaccinated mice after 14 days post-infection, whereas bacterial growth was contained in mice vaccinated with the subunit vaccine CAF01+H56 and with BCG in both lung and spleen as expected (Figures 4C,D). We found that vaccination with the subunit vaccine in presence of RA led to a significant containment of the bacterial load in the lungs 14 days after Mtb infection as compared to mice vaccinated with the subunit vaccine in the absence of RA or with BCG. Similar CFU experiments are shown on Figure S1 in Supplemental Materials.

### Pro-Inflammatory Cytokines in Lungs of Mice Immunized With CAF01+H56 in Presence of RA After Mtb Infection

In an attempt to evaluate the inflammatory response in mice vaccinated with CAF01+H56 in presence or absence of RA, or in control mice, we analyzed the cytokine and chemokine production in lungs 24 h and 14 days after Mtb infection. Strong production of pro-inflammatory cytokines, including IFNγ, IL-17, IL-1β, and Mip1β, was found in mice immunized with CAF01+H56 24 h after infection by an ELISA assay performed directly on the homogenates of the lungs. Interestingly, IFNγ and IL-17 were found to be further increased in mice immunized with the subunit vaccine in presence of RA (Figure 4E). Naive mice treated only with RA or mice vaccinated with BCG showed low levels of the cytokines. At day 14 after infection, the levels of IFNγ, and MIP1β in lung homogenates were higher in naïve and in RA treated unvaccinated mice as compared to either vaccine subunits or BCG vaccinated mice (Figure 4F), reflecting the higher bacterial load found in these groups of mice. IL1β was higher in mice that received the subunit vaccine compared to BCG suggesting that the mechanisms through which the vaccine subunits or the BCG acts are different.

All together these findings indicate that parenteral immunization with Mtb subunit vaccine in presence of RA allows the migration of Ag-specific T cells to the lung favoring a prompt pro-inflammatory microenvironment when the mycobacteria invade the lung leading to a better containment of the bacteria growth after 14 days.

### Effect of the RA Treatment on Long Lasting Protection Against Mtb Infection After Immunization With CAF01 and H56

**Figure 5:**
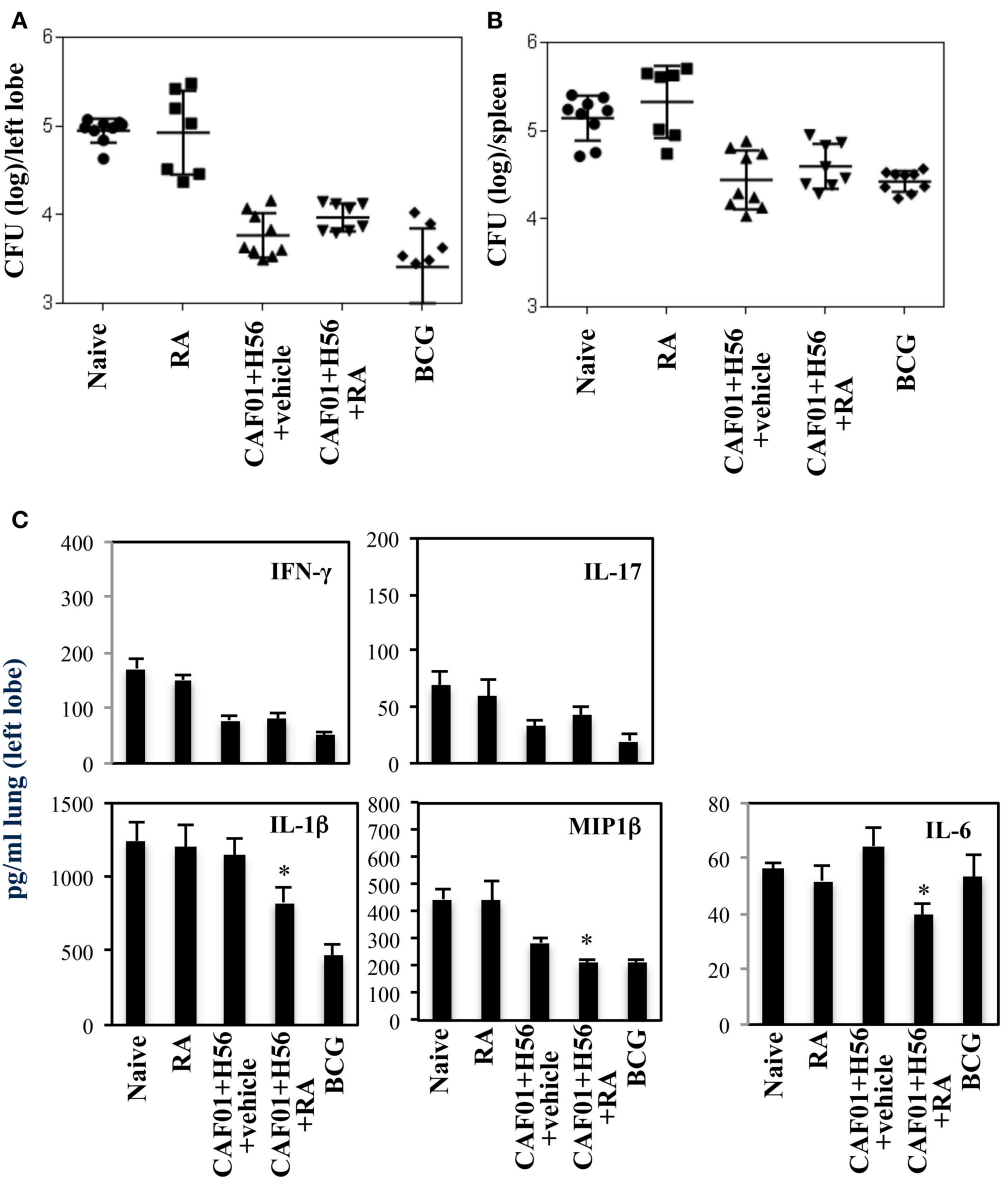
Colony forming unit (CFU) in the lung **(A)** and in the spleen **(B)** of CB6F1 mice 6 weeks after Mtb infection. Mice (7–9/group) were treated or untreated with RA (300 μg/dose, 200 μg/dose and 100 μg/dose) or its vehicle, parenterally immunized with H56 (0.5 μg/dose) and CAF01 (125 μg/dose) and infected with Mtb (10^5^ CFU) 1 month after last immunization. Colony forming units were enumerated 6 weeks after challenge. Results are expressed in log_10_ of the number of CFU/ml. Bars indicate SEM. Pro-inflammatory cytokine production **(C)** in the lung homogenates of CB6F1 mice 6 weeks after Mtb infection. IFNγ, IL-17, IL-1β, MIP1β and IL-6 levels were analyzed by ELISA assay in the lung homogenates and stars (*) indicate that the differences between the group treated with RA and the group treated with its vehicle are significant (*p* < 0.05). One rapresentative experiment is shown of three performed.

The effect of RA treatment on the protection against Mtb infection was evaluated at a later time point after infection. Mice vaccinated in presence or absence of RA, were infected with a virulent strain of Mtb (H37Rv) (10^5^ CFU/dose) 1 month after last immunization. Lung colonization by Mtb and bacterial growth were analyzed by evaluating the colony forming unit (CFU) in the lung and spleen 6 weeks after Mtb infection. Vaccination with BCG (5 × 10^5^ CFU/dose) performed once at beginning of the immunization schedule was included as control. We found that with progression of the disease 6 weeks after infection, the protection of mice vaccinated with CAF01+H56 in presence or in absence of RA was comparable in the lungs and spleens (Figures 5A,B and Figure S2 of the Supplemental Materials). On the other hand, IFNγ and IL-17 levels in lung homogenates were higher in naïve and in RA treated unvaccinated mice as compared to either vaccine subunits or BCG vaccinated mice, reflecting the higher bacterial load found in these groups of mice. The levels of IL1β, MIP1β, and Il-6 were high in unvaccinated mice and in mice that received the subunit vaccine in the absence of RA, whereas they were significantly lower in mice vaccinated with CAF01+H56 in presence of RA or with BCG (Figure 5C). This data suggest that the immune responses induced by vaccination in presence of RA had an impact on the containment of bacterial growth at early stage. In addition, even though the bacterial growth in mice vaccinated in the absence or in the presence of RA is comparable at later time points, treatment with RA during immunization is able to better contain the inflammatory response by the host.

### Higher Percentage of Mtb-Specific CD4^+^PD1^+^ T Lymphocytes in Lungs of Infected Mice Vaccinated With CAF01+H56 in Presence of RA

**Figure 6:**
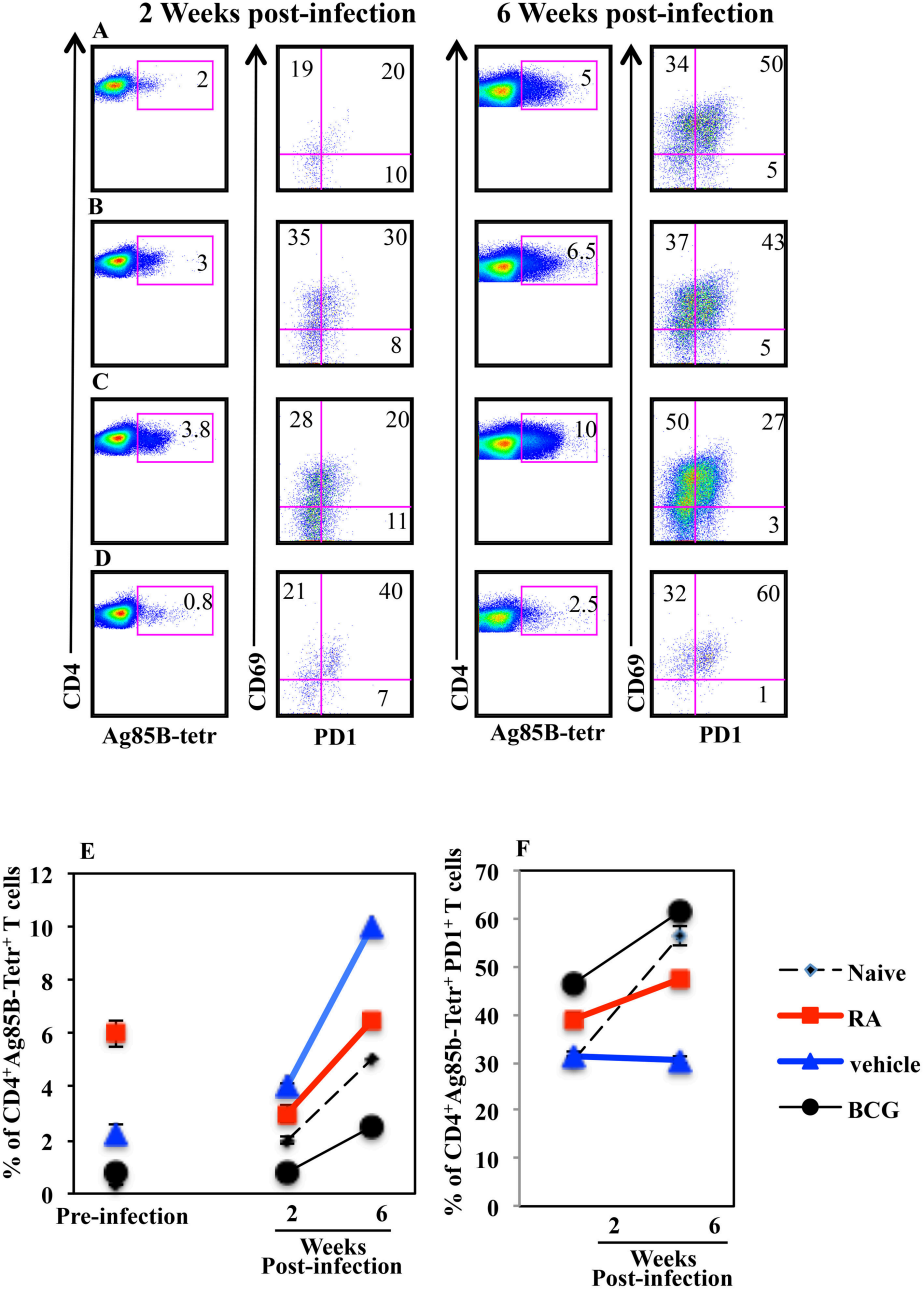
Higher percentage of Mtb specific CD4^+^PD1^+^ T lymphocytes in the lungs of infected mice immunized with CAF01+H56 in presence of RA. Untreated naïve CB6F1 mice **(A)** or mice treated with RA **(B)** or its vehicle **(C)** were immunized with CAF01 (125 μg/dose) and H56 (0.5 μg/dose) or immunized with BCG **(D)** and infected with Mtb 1 month after last immunization. Cells from the lungs were stained with anti-CD4^+^, Ag85B^+^-tetramers and anti-PD1 after 2 (left) and 6 (right) weeks after the infection. The graphs show the percentage of CD4^+^Ag85B^+^ cells at pre-infection and 2 and 6 weeks after infection **(E)** and the percentage of CD4^+^Ag85B^+^PD1^+^ T cells 2 and 6 weeks after infection **(F)**. Bars indicate the SEM. One rapresentative experiment is shown of two performed.

Next, to evaluate the correlation between progression of the Mtb infection and the presence of Mtb specific T lymphocytes in the lungs of mice vaccinated in presence or absence of RA, we analyzed the Ag85B specific T lymphocytes 2 and 6 weeks post infection by flow cytometry. We found that the percentage of Ag85B-specific T cells increased in all groups of mice following Mtb infection including vaccinated and unvaccinated mice (Figure 6). Mice vaccinated with the subunit vaccine CAF01+H56 in presence of RA, which had a higher percentage of Ag85B tetramer specific T lymphocytes before infection, at 2 and 6 weeks post-infection showed a reduced percentage of Ag-specific T lymphocytes as compared to CAF01+H56 vaccinated mice in presence of RA vehicle (Figures 6B,C). In addition, a higher percentage of Ag85B tetramer specific T lymphocytes of mice vaccinated with the subunit vaccine in presence of RA, expressed PD1 molecules as compared to mice vaccinated with CAF01+H56 in presence of RA vehicle (Figures 6B,C), suggesting that an exhaustion following activation occurred in Ag-specific T cells in the lung of mice vaccinated in presence of RA. On the other hand, both naïve and BCG vaccinated mice had lower Ag85B tetramer positive cells in the lung, which express high levels of PD1 molecule (Figures 6D,F), suggesting that the mechanisms underlying the containment of the bacteria growth in the lung of mice vaccinated with the subunit vaccine or with BCG are different. These data suggest that a higher percentage of Ag85B positive T lymphocytes colonizes the lung when mice are immunized with the CAF01+H56 vaccine in presence of RA and that the immunization protocol provides an early but transient protection to Mtb infection.

## Discussion

Safe and effective mucosal adjuvants to be delivered with purified recombinant proteins or pathogen' subunits are not yet available. Here, we investigated the ability of RA to modulate antigen-specific induced immune responses at mucosal sites. We hypothesized that this vaccine strategy could induce mucosal immune responses in the absence of mucosal delivery by using commercially available systemic adjuvants. Few substances have been demonstrated to be effective as mucosal adjuvants in experimental animal model, which include cholera toxin (CT) and the Escherichia coli heat-labile enterotoxin (LT) (2, 11, 12, 29). However, because of the toxicity of CT, LT and of their derivatives and because of the side effects reported after intranasal delivery of an LT mutant, the clinical use of these molecules is not recommended (8–10). Here, we found that parenteral vaccination with CAF01+H56 in presence of RA induces mucosal H56-specific IgA responses and an enhanced percentage of Ag-specific CD4^+^ T lymphocytes homing to the lungs. CAF01 is a systemic adjuvant, which is able to shape the immune response toward Th1 and Th17 cells when delivered with different types of Ag (30–33), but unless CAF01 and Ag are delivered through mucosal route following parenteral immunization or given by simultaneous subcutaneous and nasal immunization, it does not induce strong mucosal immunization (34, 35). Therefore, by using RA in combination with CAF01 or with currently employed systemic adjuvants it could be of advantage to redirect the immune responses to mucosal tissues. Retinoic acid promotes mucosal immunity by inducing the expression of mucosal homing receptors α4β7 and CCR9 on T and B lymphocytes (13, 19, 20). The effect of RA on B cells leads to the isotype switching of IgA antibodies (19, 36–39). This is in accordance with our findings, indeed higher OVA and H56 specific IgA and specific IgA secreting long living plasma cells were induced in mice after parenteral immunization with CAF01+H56 with RA, as compared to control mice. Furthermore, RA promotes humoral and cellular immunity at both mucosal and peripheral tissues (40). Indeed, in the absence of RA-mediated signaling, defective T cell activation and differentiation occur at mucosal and in peripheral sites (13–16, 41–43). In addition, RA is required for the homeostasis and differentiation of splenic and mucosal dendritic cells (17, 18, 44). Because of these effects the higher susceptibility to infection and the low response to vaccination in subjects with low vitamin A intake (41–45) could be explained. Although RA is primarily implicated in gut homing, it has been described that it also increased the accumulation of vaccine specific T cells at other mucosal sites including the vagina and lungs (21). However, the mechanisms for the homing to the extra-intestinal mucosa tissues have not been fully evaluated.

Tuberculosis (TB) is a life threating diseases and the TB vaccine used today, the Bacillus Calmette-Gueren (BCG), provides limited protection against TB for new-borns, children and adults, which accounts for most of the TB cases worldwide (46). Therefore, new candidate vaccines against TB are highly needed and some of them are under evaluation in clinical trials (47). Vaccine-induced protection against pulmonary tuberculosis (TB) is still missing and it has been associated to a rapid recruitment of Mtb-reactive Th1 cells in the lungs (48–50). Indeed, it has been observed that Mtb has the ability to infect the lungs while delaying the onset of adaptive immune responses (49–52). It has been described that immunization with the tuberculosis subunit vaccine CAF01+H56 promotes lung homing of effector CD4^+^ T cells (23). Here, we found that parenteral vaccination with CAF01+H56 in presence of RA further enhances the migration of Ag-specific T lymphocytes in both parenchyma and airway lung compartments. Therefore, as a next step we analyzed the effects of RA treatment after challenge with virulent Mycobacterium tuberculosis (Mtb) strain after parenteral vaccination with the CAF01+H56 subunit vaccine. Immunization with CAF01+H56 in presence of RA results in a lower bacterial loads in the lungs of mice, 14 days after challenge with virulent Mycobacterium tuberculosis (Mtb) as compared to mice immunized in the absence of RA or vaccinated with BCG. On the other hand, no differences were observed in the bacterial loads measured in the spleens in mice treated or not with RA. Therefore, it could be argued that the recruitment of Ag-specific T cells and the immune responses elicited at mucosal sites after vaccination in presence of RA could be related to the containment of the bacterial growth in the lungs. Furthermore, higher pro-inflammatory cytokines were found in the lungs of mice immunized with CAF01+H56 in presence of RA 24h after Mtb infection as compared to unvaccinated and BCG vaccinated mice. In particular, in mice vaccinated with CAF01+H56 in presence of RA higher levels of IFNγ and IL-17 were detected. At day 14 after infection, the levels of IFNγ and Mip1β in lung homogenates were higher in naïve or in RA treated unvaccinated mice as compared to either vaccine subunits or BCG vaccinated mice, reflecting that the higher bacterial load found in these groups of mice is proportional to the pro-inflammatory cytokine production (53). However, with the progression of the disease, 6 weeks after infection, bacteria growth in mice vaccinated with CAF01+H56 in presence or in absence of RA was comparable, suggesting that the immune responses induced after vaccination in presence of RA was able to contain bacterial growth at early stages. These data are in agreement with others reporting that RA is effective to increase vaginal immune response only after the first dose of vaccine and it failed to expand T cells upon re-exposure to the antigen (54), suggesting that RA may have acted or induced preferentially terminally differentiated T cells. Of note, even if the bacterial growth was comparable at a later time point, treatment with RA during immunization was able to better contain the inflammatory response by the host. Indeed, the levels of IL-1β, MIP1β, and IL-6 pro-inflammatory cytokines are lower in mice vaccinated with CAF01+H56 in presence of RA treatment as compared to mice immunized with the subunit vaccine in the absence of RA, suggesting that RA treatment limits the damage caused by an excessive immune response to the infection. Furthermore, by analyzing Ag85B tetramer specific T lymphocytes in the lungs after 2 and 6 weeks post-infection, we observed a contraction of Ag-specific T lymphocytes in mice vaccinated with CAF01+H56 in presence of RA and an increase of Ag85B-specific T lymphocytes expressing PD1 molecules as compared to mice vaccinated with CAF01+H56 in presence of RA vehicle, suggesting that an exhaustion following activation occurred in Ag-specific T cells in the lungs of mice vaccinated in presence of RA. However, it has been reported that the inhibition of the immune response mediated by PD1 is essential in the control of pulmonary Mtb infection and that PD1 mediated suppression is required to prevent the detrimental over production of pro-inflammatory cytokines, which leads to the death of the host (55, 56). Indeed, PD1 deficient mice are extraordinarily sensitive to tuberculosis (57). On the other hand, both naïve and BCG vaccinated mice had lower Ag85B tetramer positive cells in the lung, which express high levels of PD1 molecule, suggesting that the mechanisms underlying the containment of the bacteria growth in the lung of mice vaccinated with the subunit vaccine or with BCG are different. These data show that an enhanced mucosal immune response is generated during parenteral vaccination in presence of RA, that RA treatment leads to better containment of the bacteria growth at early stages and concurs to prevent host damage by limiting the pro inflammatory cytokines and inducing a higher percentage of PD1^+^ Ag-specific T cells in the lungs. Although the effect of RA on vaccination against TB in term of protection is transient, its effect in delaying bacterial growth and limiting tissue damage could be an advantage for the host. Therefore, focusing on RA in developing new vaccines against pathogens, that invade through mucosal routes and cause either chronic or acute infection should be considered and further investigated.

## Ethics Statement

Animals were maintained under specific pathogen-free conditions in the animal facilities at the Istituto Superiore di Sanità (ISS) and treated according to European Union guidelines and Italian legislation (Decreto Legislativo 26/2014). All animal studies were authorized by the Italian Ministry of Healthy and reviewed by the Service for Animal Welfare at ISS (Authorization n. 82014-b of 15/01/2014). All animals were euthanized by CO2 inhalation using approved chambers, and efforts were made to minimize suffering and discomfort.

## Author Contributions

SV designed the experiments, analyzed the data, and wrote the article. AR, GP, and CP performed experiments analyzed the data and critically edited the manuscript. DC and PA provided key reagents and critically edited the manuscript. All authors have contributed to the drafting of the manuscript, have revised the work, and have approved the final version.

### Conflict of Interest Statement

The authors declare that the research was conducted in the absence of any commercial or financial relationships that could be construed as a potential conflict of interest.
