# Supplementary material for: Parenteral Vaccination With a Tuberculosis Subunit Vaccine in Presence of Retinoic Acid Provides Early but Transient Protection to M. Tuberculosis Infection
Source: Front Immunol. 2019 May 3;10:934. doi: 10.3389/fimmu.2019.00934 (PMC6509564; doi:10.3389/fimmu.2019.00934)
Supplement: Supplementary file 1 [file Data_Sheet_1.doc]

**Figure 1.** Colony forming unit (CFU) in the lung (ACEG) and in the spleen (BDFH) of CB6F1 mice. Mice (3-5/group) were treated or untreated with RA (300 μg/dose, 200 μg/dose and 100 μg/dose) or its vehicle, parenterally immunized with H56 (0.5 μg/dose) and CAF01 (125 µg/dose) and infected with Mtb (10^5^ CFU) one month after last immunization. Colony forming units were enumerated 24 hours and 14 days in the lung (ACEG) and in the spleen (BDFH) after challenge. Results are expressed in log_10_ of the number of CFU/ml. Bars indicate SEM and stars (*) indicate that the differences between the group treated with RA and its vehicle are significant (p<0.05). Two independent experiments are shown.

**Figure 2.** Colony forming unit (CFU) in the lung (AC) and in the spleen (BD) of CB6F1 mice 6 weeks after Mtb infection. Mice (3-5/group) were treated or untreated with RA (300 μg/dose, 200 μg/dose and 100 μg/dose) or its vehicle, parenterally immunized with H56 (0.5 μg/dose) and CAF01 (125 µg/dose) and infected with Mtb (10^5^ CFU) one month after last immunization. Colony forming units were enumerated 6 weeks after challenge. Results are expressed in log_10_ of the number of CFU/ml. Bars indicate SEM. Two independent experiments are shown.
